# Birds vs bricks: Patterns of species diversity in response to urbanization in a Neotropical Andean city

**DOI:** 10.1371/journal.pone.0218775

**Published:** 2019-06-20

**Authors:** Juan David Carvajal-Castro, Ana María Ospina-L, Yemay Toro-López, Anny Pulido-G, Laura Ximena Cabrera-Casas, Sebastián Guerrero-Peláez, Víctor Hugo García-Merchán, Fernando Vargas-Salinas

**Affiliations:** 1 Grupo de investigación en Evolución, Ecología y Conservación (EECO), Programa de Biología, Universidad del Quindío, Armenia, Colombia; 2 Instituto de Investigación de Recursos Biológicos Alexander von Humboldt, Bogotá, Colombia; University of Michigan, USA

## Abstract

Urbanization is currently one the most important causes of biodiversity loss. The Colombian Andes is a well-known hotspot for biodiversity, however, it also exhibit high levels of urbanization, making it a useful site to document how species assemblages respond to habitat transformation. To do this, we compared the structure and composition of bird assemblages between rural and urban habitats in Armenia, a medium sized city located in the Central Andes of Colombia. In addition, we examined the influence of urban characteristics on bird species diversity within the city of Armenia. From September 2016 to February 2017 we performed avian surveys in 76 cells (250 x 250 m each) embedded within Armenia city limits; and in 23 cells (250 x 250 m each) in rural areas around Armenia. We found that bird diversity was significantly lower in urban habitats than in rural habitats, and differed in species composition by 29%. In urban cells, with higher abiotic noise intensity and higher impervious surface area, we found lower bird diversity than that in urban cells with higher guadual (*Guadua angustifolia* patches), and forested surface areas. We did not find segregation of urban cells according to the species composition, although additional bird surveys inside urban forests remnant are needed to be more conclusive about this aspect. Altogether, our results highlight the importance of green areas embedded within cities to conserve bird diversity through reducing the ecological impact of urbanization on avian biodiversity.

## Introduction

Urbanization is one of the most important causes of environmental transformation, reducing natural habitats and replacing them with impervious surfaces [1, 2]. Urban areas worldwide share many features such as high levels of chemical, visual, and acoustic pollution, high human population densities, and the predominance of simple anthropogenic structures. Therefore, urbanization usually causes a general loss of biodiversity and promotes biotic homogenization, that is, a reduction in the turnover of species (beta diversity) among assemblages [3, 4, 5]. In addition, urbanization promotes alteration of natural diversity distribution patterns [6], and a reduction in the phylogenetic and functional diversity of local biotic communities with the concomitant effects on loss of the evolutionary history of lineages and ecosystem services related to human well-being [7, 8, 9].

Studies on the effects of urbanization at the assemblage level have been related mainly to species richness and the abundance of individuals per species [10, 11], and according to the scale of the study, some patterns are consistently observed. For instance, in studies comparing urban versus rural habitats, it is common to find a significant reduction in species richness in the urban habitats [1, 12]. Also, studies covering an urban-rural gradient have documented that diversity of species frequently increases at intermediate levels of urbanization [4, 12], while studies comparing different land coverages within a city, show that species richness is inversely correlated to impervious surface area [13, 14, 15]. Species composition also shows specific patterns in urban habitats [1, 16]. While well conserved rural areas are dominated by urban avoiders (e.g. species of forest interior), places with intermediated levels of urbanization are dominated by species adapters (e.g. edge forest species), and in highly urbanized places, urban exploiters are most common (commensals, species highly efficient or dependent on exploiting resources provided by humans) [16, 17].

Most of the studies about the effects of urbanization on species diversity have been carried out in temperate zones [2, 10, 11, 18, 19, 20, 21, 22], while in the tropics, especially in the Americas, the research has been sparse [15, 23, 24, 25]. This bias in research efforts is concerning because patterns observed in temperate regions can often be hard to generalize to poorly studied regions in the tropics [15]. This is, in part, because the effect of urbanization on species diversity depends on the urban developmental dynamics, and on the ecology of ecosystems and organisms within a given region [26, 27]. Moreover, tropical regions exhibit higher species diversity than temperate regions [28]; hence, the effect of urbanization on species represented only in tropical communities could be missing from temperate area generalizations [29, 30].

Birds have long been used to study the effects of urbanization on species diversity [9, 15, 31, 32]. This is because they exhibit a wide range of behaviors, they can be locally abundant, easily observed, and appropriate taxonomic identification is feasible for many species in the field. Additionally, there have been reports of both negative and positive effects derived from urbanization on birds, which makes them ideal models for comparative studies [31, 32]. Negative effects on bird species diversity are associated with the natural habitat transformation that causes a reduction in resources like nesting sites and food availability [33]. Additionally, many birds die due to window collisions [34], and exposure to novel diseases [35] and predators [36, 37]. However, some species are positively affected by urbanization because they are pre-adapted and some of them take advantage of resources associated with anthropogenic environments, such as novel food, shelter, and/or nest sites [38–40].

In the last two decades, some studies were conducted using birds in urban habitats of Latin America [14, 15, 25, 41, 42, 43, 44], but not all of them were in tropical regions. Thus, there is a gap in knowledge which still exists about the structure (i.e. richness and relative abundance of species) and composition of Neotropical bird assemblages in towns and cities. Many questions remain unanswered or need to be evaluated based on more empirical evidence. For instance, how much does the structure and composition of species assemblages change in gradients of natural environments to urbanized areas like cities and towns? How might species diversity change spatially among places with different levels of urbanization within a city? What is the relative importance of factors associated to urbanization level (e.g. intensity of anthropogenic noise, amount of impervious surface) in determining the distribution of species? And how might the presence or implementation of urban forest remnants benefit the maintenance of high species diversity within cities?

The Colombian Andes is one of the most diverse regions in Latin America and on the planet, with many species exhibiting limited geographical ranges of distribution, and with evolutionary processes leading to speciation occurring at particular high rates [45, 46, 47]. However, this region also exhibits a high level of urbanization [48]. We studied the diversity of birds in and around a medium-size city located in the Central Andes of Colombia to answer questions about the effects of urbanization in the structure and composition of vertebrate communities in tropical regions of Latin America. First, we compared bird assemblages between urban and rural habitats, expecting to find that the latter habitat exhibits higher species diversity than the former and that both differ in species composition. Second, we tested the relationship between urban and rural habitat characteristics (e.g. anthropogenic noise intensity, amount of impervious surface) and the structure of bird assemblages; we predicted that sites with high levels of urbanization would exhibit low species diversity. Third, we examined whether species composition in bird assemblages reflects urbanization level; if this is the case, we expected to find similar species compositions between sites with similar habitat characteristics. To our knowledge, this is the first study in Colombia (but see [49]), and one of the few in the Neotropics, which quantifies the relationship between spatial variation in bird diversity and urban habitat characteristics.

## Materials and methods

### Ethics statement

Procedures for the observation of birds in fieldwork followed all the ethical requirements and were according to the permits 374 of March 2014 and 240 of February 2018 given by the Corporación Autonóma Regional del Quindío (CRQ), Colombia.

### Study area

**Fig 1:**
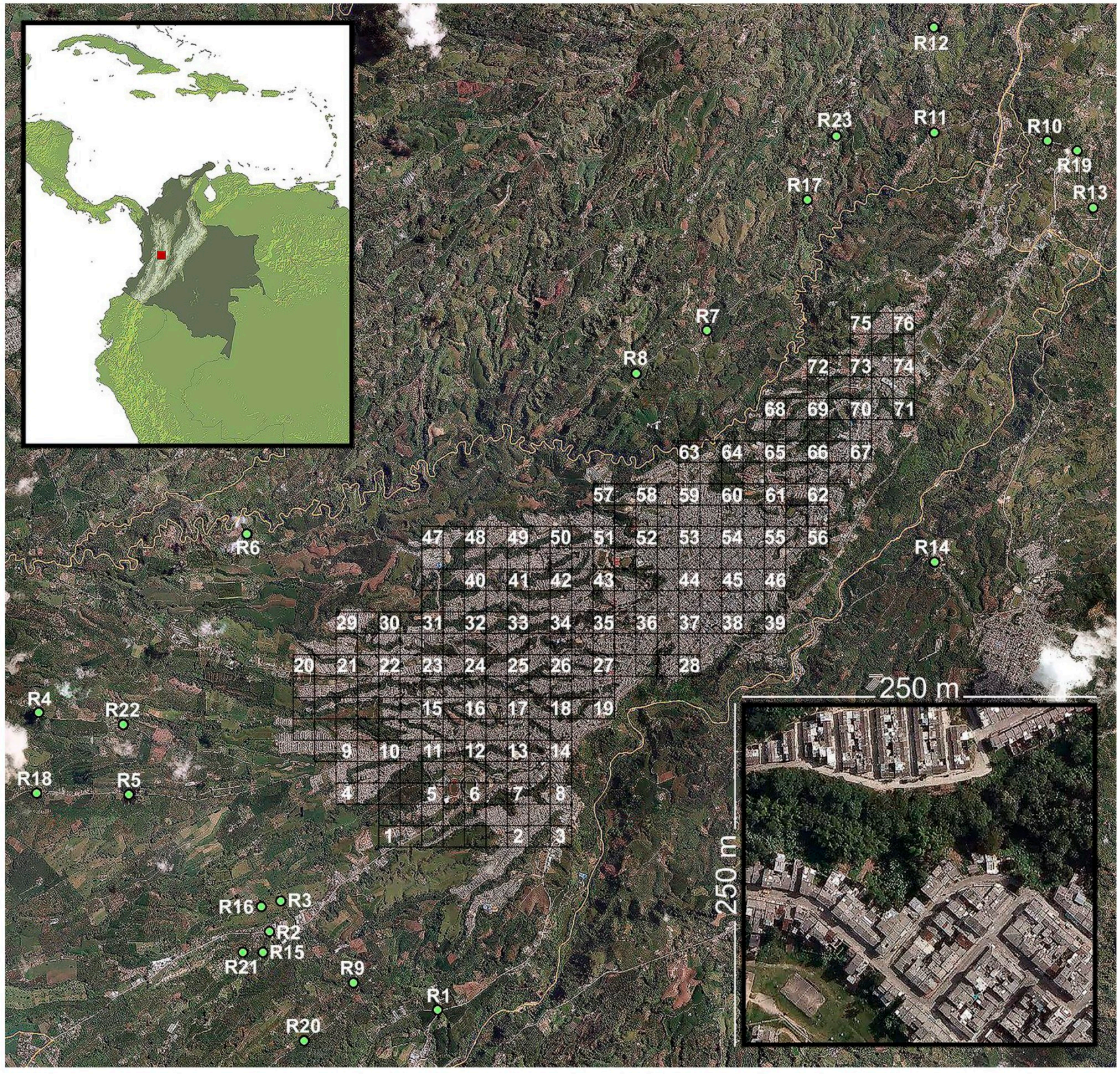
Upper left panel: Geographic location of the study area. Central panel: image of the city of Armenia, Central Andes of Colombia, South America, showing the grid designed to select urban cells (250 m x 250 m each). Numbers within grid point urban cells indicate where habitat characteristics and bird surveys where performed (Sept 27 of 2016 –Feb 17 of 2017), while green dots and respective code indicate sampled rural cells (Nov 29 of 2016 –Feb 17 of 2017). Lower right panel: example of one of the 76 urban cells where different types of coverages are present (i.e. forest, guadual, and open and impervious surface areas). The yellow line indicates the political limits of the municipality of Armenia. Image taken from Sig-Quindío (CRQ-GIS 2017).

Armenia, a medium size city in the Central Andes of Colombia (4.516667°N, 75.66666°W; 1483 m elev.), offers a good opportunity to answer questions about the effects of urbanization on species diversity and composition because it is located in a region characterized by its high biodiversity [50], and because it encompasses places with high levels of urbanization (i.e. predominance of buildings and impervious surfaces), and others with predominantly green areas (i.e. forest remnants and guaduals) (Fig 1).The average annual temperature of Armenia is 22°C with a relative humidity above 80%; precipitation is seasonal, with approximately 2119 mm/year rainfall [51]. Armenia comprises an area of 23.57 Km^2^ and has >290,000 habitants [52]. It is divided by several biological corridors composed by remnant forests and Neotropical giant bamboo *Guadua angustifolia* (guadual) patches [53]. These biological corridors embedded in the urban matrix represent approximately 30% of the total area of the city [54]. Armenia is located in a landscape with some isolated remnant forests embedded in a matrix of grasslands used for livestock and agriculture (Fig 1). The composition and structure of vegetation in forest remnants inside of Armenia seems to be similar among them and with respect to forest remnants in surroundings areas of the city [55, 56, 57].

### Bird urban surveys

To establish our urban sampling units for bird surveys, we divided the city of Armenia in 364 cells of 250 m x 250 m each (hereafter urban cells). We created the urban cells using a satellite photograph of Armenia obtained from SIG-Quindío [54], and by overlapping a grid using the Fishnet application with the software ArcGis 10.1 (ESRI). From all the urban cells in our grid we chose one randomly as a starting point. We then selected other cells systematically (in different cardinal directions) using the criterion that they were separated each other by at least 500 m (i.e. two urban cells apart). We surveyed urban cells separated by 500 m to reduce the chances of re-counting an individual bird more than once.

We performed a standardized technique for bird surveys according to recommendations in [58]. We made bird counts only on sunny days between September 27^th^ 2016, and February 17^th^ 2017. Surveys were conducted by two observers with more than two years of bird survey experience; they used Celestron outland X 10x42 binoculars to sample 1–4 urban cells between 0600 h– 0800 h each day. Each urban cell was sampled only once during the whole study. After arriving to the center of each urban cell, we established two orthogonal transects of 100 m each; one of these transects was randomly selected for the avian surveys which lasted 20 minutes, and were performed always observing forward to reduce the chance of double-counting. We recorded species richness and number of individuals per species. During the observation period for each cell, we only included birds observed within a distance < 50 m from the central line of the transect; we did not include individuals flying over the sampling cells. We also excluded counts of any migratory species observed. Because anthropogenic noise level varies among urban cells and hence, the detectability of bird songs by researches, we based our bird counts only on visually observed individuals in order to avoid a bias in counts between sites [59].

Adjusting for detectability in birds surveys is important before performing spatial or temporal comparisons [55]. However, we did not perform analyses on detectability because standardized non-detectability-based counts (like ours) provide adequate information on community structure and relative abundances of birds in urban areas [60, 61]. Moreover, our maximum observation distance was 50 m, which is a commonly used threshold for bird counts and does not cause reductions in detectability when compared with smaller sampling distances [62]. Like [15] we assumed that possible biases (except by noise level) associated with bird detectability were constant and distributed throughout all urban cells. All bird species in our study area were easily recognizable and identified using the field guides by [63] and [64]. We followed the taxonomical identification proposed by [65], and the threatened status of the species was recorded according to [66] and [67] and the IUCN [68].

### Urban habitat characteristics

In the satellite image, we measured habitat characteristics that could influence species richness and evenness for each urban cell where we performed bird surveys. We measured straight distances to the nearest boundary of the city, percentage of impervious surface area, percentage of guadual surface area, percentage of forested surface area, and percentage of open area (e.g. grasslands, soccer fields). We estimated percent coverages using the function *polygons* in QGIS 2.16 [69]. In addition, for each urban cell, we measured the anthropogenic noise intensity (hereafter noise) using a sound meter Extech 407730 (measured at the middle of each bird survey). Since noise intensity was measured in decibels (dB), which represent a logarithmic scale, the calculation of average values and other statistical analyses were conducted after converting dB values to a linear scale (pressure, Pa).

### Bird rural surveys

We used the same satellite image as for the establishment of urban cells to randomly choose 250 x 250 m rural plots (hereafter rural cells) outside the urban boundary of Armenia (Fig 1). However, only was possible to perform bird surveys in a sample of the pre-selected rural cells due to restrictions imposed by the absence of secure paths and private land owners. Habitat characteristics were measured using the same software tools mentioned for urban cells, but in rural places we included measures of percentage of cultivated areas (mostly by coffee) which were absent from urban cells. The level of anthropogenic noise was not measured in each rural cell, but it was clearly lower than in urban cells. Bird surveys in rural cells were made between November 29^th^ 2016, and February 17^th^ 2017, and conducted using the same methods as described for urban cells. Bird surveys in rural cells were made from open areas (i.e. grasslands), and similar than in urban cells, we did not perform bird surveys inside forested areas.

### Data analysis

We used diversity indexes proposed by [70] and [71]. We made calculations of diversity with different levels of sensibility to the evenness of species (i.e. q = 0, q = 1, q = 2). When q = 0, the calculation of diversity ignores differences in the relative abundance of species and the obtained value is equivalent to species richness; when q tends to 1, species are weighted according to their relative abundance and the obtained value corresponds to the exponential of the Shannon-Wiener index; when q = 2, the obtained value is mainly influenced by the most abundant species and corresponds to the inverse of the Simpson index. To compare bird species diversity between urban and rural habitats and test completeness of our bird surveys, we performed an analysis of sampling coverage (sensu [72]) using the iNEXT package [73] in R software [74]. The sample coverage varies between 0 and 1, and it is a measure of sample completeness based on the proportion of the total number of individuals in a community that belong to the species represented in the sample. In addition, sample coverage allows to compare species diversity between places while controlling by biases existing each time that similar comparisons were made standardizing by sample size [72]. This analysis was performed pooling the abundances per species observed in all cells [75].

The similarity of species between habitats was compared with the index of beta-diversity proposed by [75], which is the ratio between Gama-diversity [D(Hϒ)] and the mean of Alpha-diversity [D(Hα)]. The advantage of this index over previous measures of beta diversity is its independence of differences in alpha values between compared habitats. If this Beta-diversity index tends to equal 1, it means that both habitats (urban and rural) are similar in species composition and structure, but if the index tends to equal 2, it means that the habitats are very different [76]. Also, to test possible patterns of similarity among urban and rural cells according to species composition, we performed a Non-metric Multidimensional Analysis (NMDS) using the vegan package [77] in R.

To test the relationship between urban and rural habitat characteristics and the structure and composition of bird assemblages, we calculated bird diversity for each urban and rural sampling cell. Because structural habitat characteristics can be intercorrelated, we decreased redundancy by conducting a Principal Component Analysis (PCA) with Varimax-rotation in the software SPSS v. 21 [78]. Then, we conducted a generalized lineal model (GLM) with the Poisson error distribution between the PCAs, noise intensity, and bird species diversity (at different q values) to test how habitat characteristics influence bird diversity. The bird species diversity data in the urban habitat were analyzed for autocorrelation using the Moran’s index. The diversity at order q = 0 did not exhibit a significant spatial autocorrelation (Moran’s index = 0.01, P = 0.08). For the diversity at order q = 1 and q = 2, the autocorrelation was significant (Moran’s index = 0.018, P = 0.03, and Moran’s index = 0.02, P = 0.01, respectively), but given that Moran’s index was small we can assume the effect in our results would be too small. In a similar way, the bird species diversity data in the rural habitat did not exhibit spatial autocorrelation at orders q = 0, q = 1 and q = 2 (Moran’s index = 0.027, P = 0.31, and Moran’s index = -0.1, P = 0.4538, Moran’s index = -0.1227, P = 0.2916, respectively). Altogether, these analyses indicate that the diversity of bird species in sampling cells close to each other are not more similar than the diversity in sampling cells located farther away. These analyses were performed in ape packages [79].

Finally, we tested possible seasonality effects in our data. For this, we included Julian days as a random effect in a GLMM, and we do not found that Julian days has an effect in our models (p-value > 0.1). Therefore, we did not include this variable in our statistics about species diversity and habitat characteristics. This analysis was performed in lme4 and RLRsim packages for R [80, 81].

## Results

**Fig 2:**
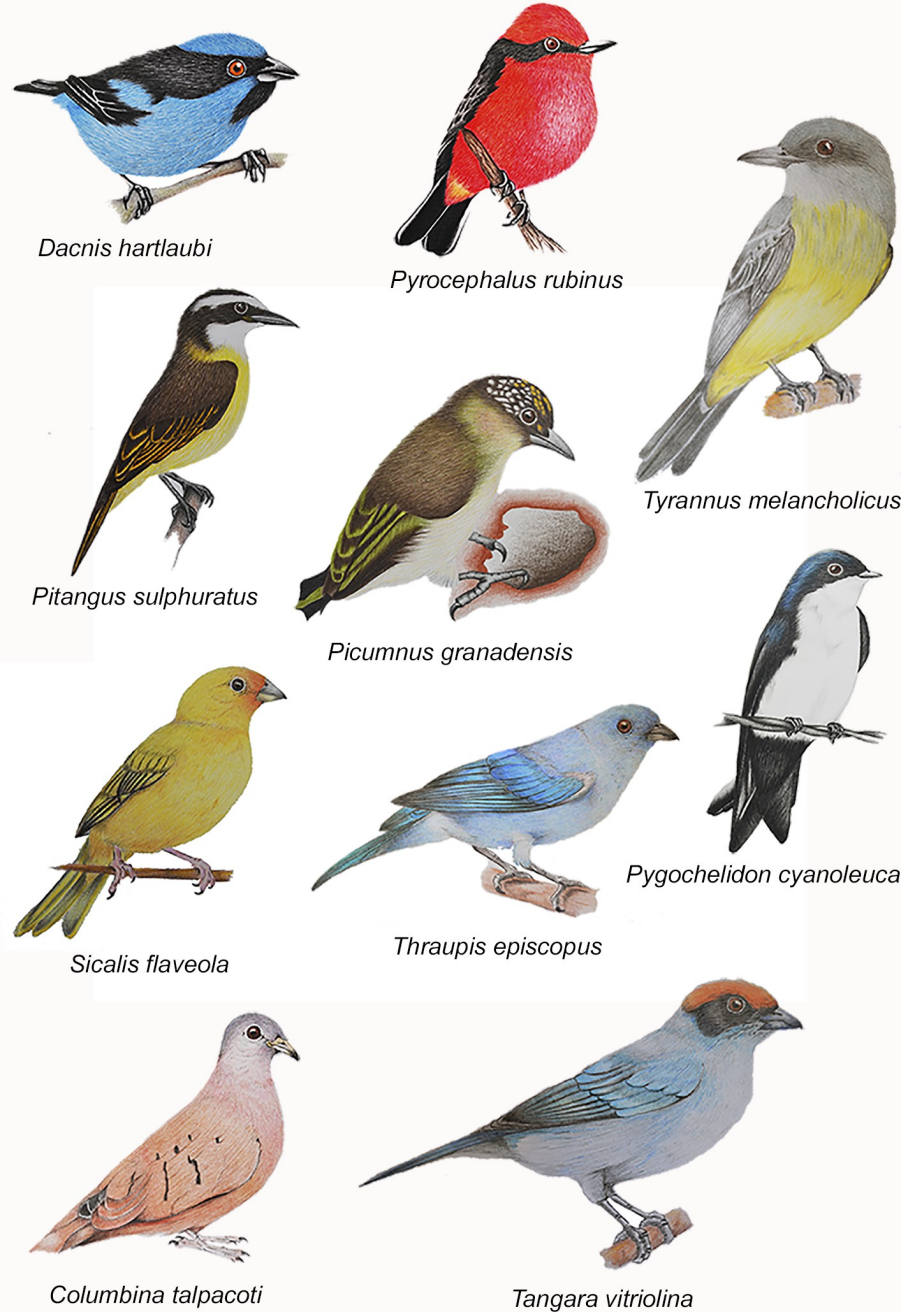
Panel showing some representative species of the bird diversity in the city of Armenia, Colombia. Species shown correspond those with most abundance of individuals in urban cells (Illustrations by Yemay Toro-López and Anny Pulido-G), and a unique species catalogued as Vulnerable by the IUCN 2017 (*Dacnis hartlaubi*).

We conducted bird surveys in 76 urban cells where we recorded 3,625 individuals belonging to 75 species (S1 Table); only one species (*Dacnis hartlaubi*) was cataloged with a status of threatened (Vulnerable *sensu* 60) (Fig 2). We surveyed 23 rural cells and recorded 1,069 individuals belonging to 85 species; none of them catalogued under a threatened category (S1 Table). Raw data about bird records underlying our findings are available at Figshare (doi: 10.6084/m9.figshare.8132132) and on request to authors.

**Fig 3:**
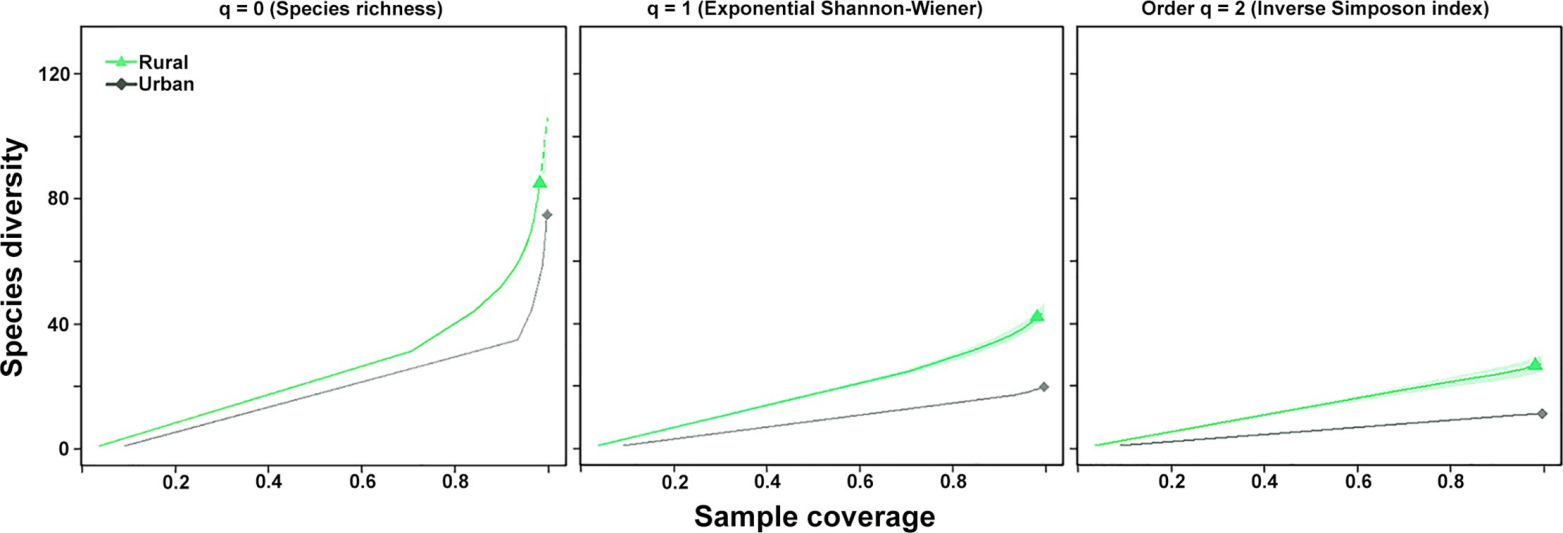
Coverage-based rarefaction (solid line) and extrapolation (dashed line) plots with 95% confidence intervals (shaded area) for bird species diversity (based on Hill numbers, q = 0, 1, 2) between urban and rural habitats. Reference samples are denoted by solid symbols.

**Fig 4:**
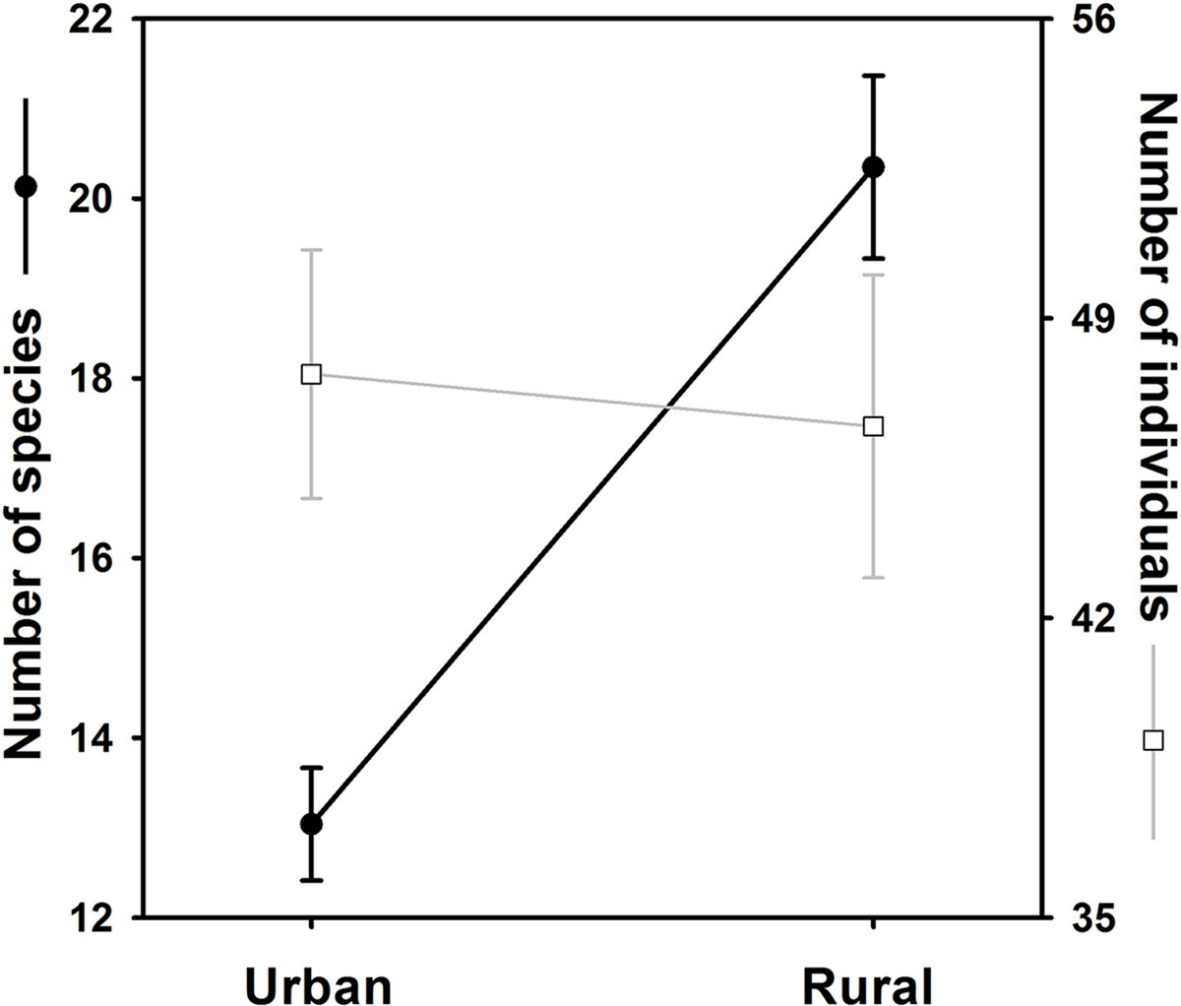
Species richness and number of individuals (abundance per sampling cell) between bird assemblages in the urban and rural habitats. Data shows mean values with standard error of the mean.

When we compared the diversity of bird assemblages in the 76 urban cells with those obtained in the 23 rural cells, we found that species diversity was much higher in the rural habitat compared to the urban habitat (Fig 3). Standardizing at a sample coverage of 0.99, the diversity of birds in the city of Armenia at order q = 1, 2, and 3, was 1.34, 2.2, and 2.45 times lower than in rural habitats, respectively. Although the number of species was higher in the rural habitat, the mean number of individuals per sampling cell did not differ between habitats (Fig 4). Moreover, the Beta-diversity index showed that the bird species composition between the two habitats differed only around a 29%: D (Hβ) = 1.29.

**Fig 5:**
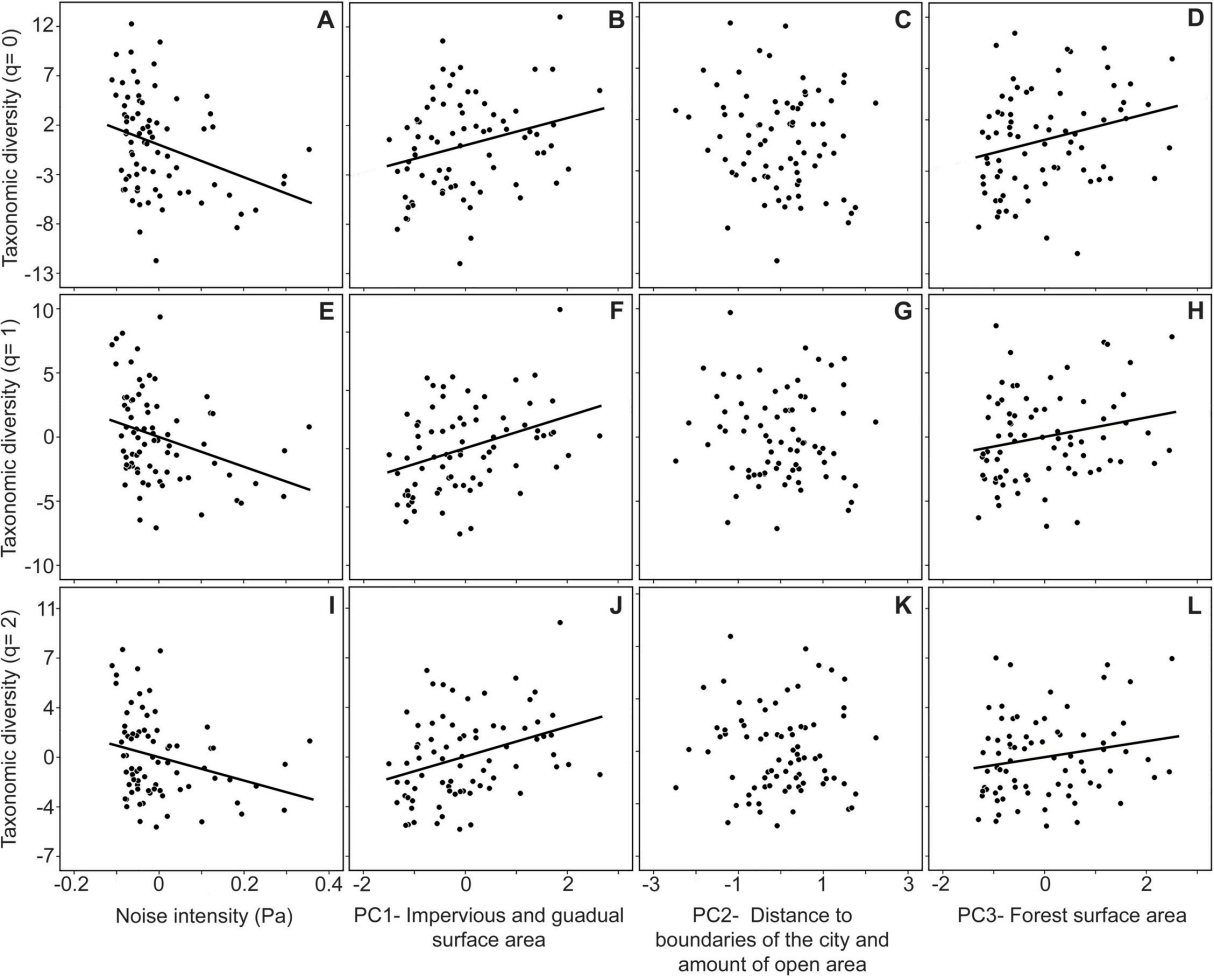
Partial plots of a multiple regression analysis showing the Relationship between the bird species diversity at different q orders (i.e. 0,1,2) and the intensity of noise (A,E,I), the percentage of impervious surface and guadual surface area (B,F,J), distance to the edge of the city and percentage of open surface area (C,G,K), and percentage of forest surface area (D,H,L) in 76 urban cells in the study area This figure has illustrative aim due that results and discussion are based on results obtained in a Generalized lineal model(GLM), however, results obtained in the GLM and the multiple regression analyses were similar. Values on *y*-axis (left) and *x*-axis (bottom) are residuals of a multiple regression analysis. Regression lines in bold indicate significant relationships at alpha = 0.05.

**Fig 6:**
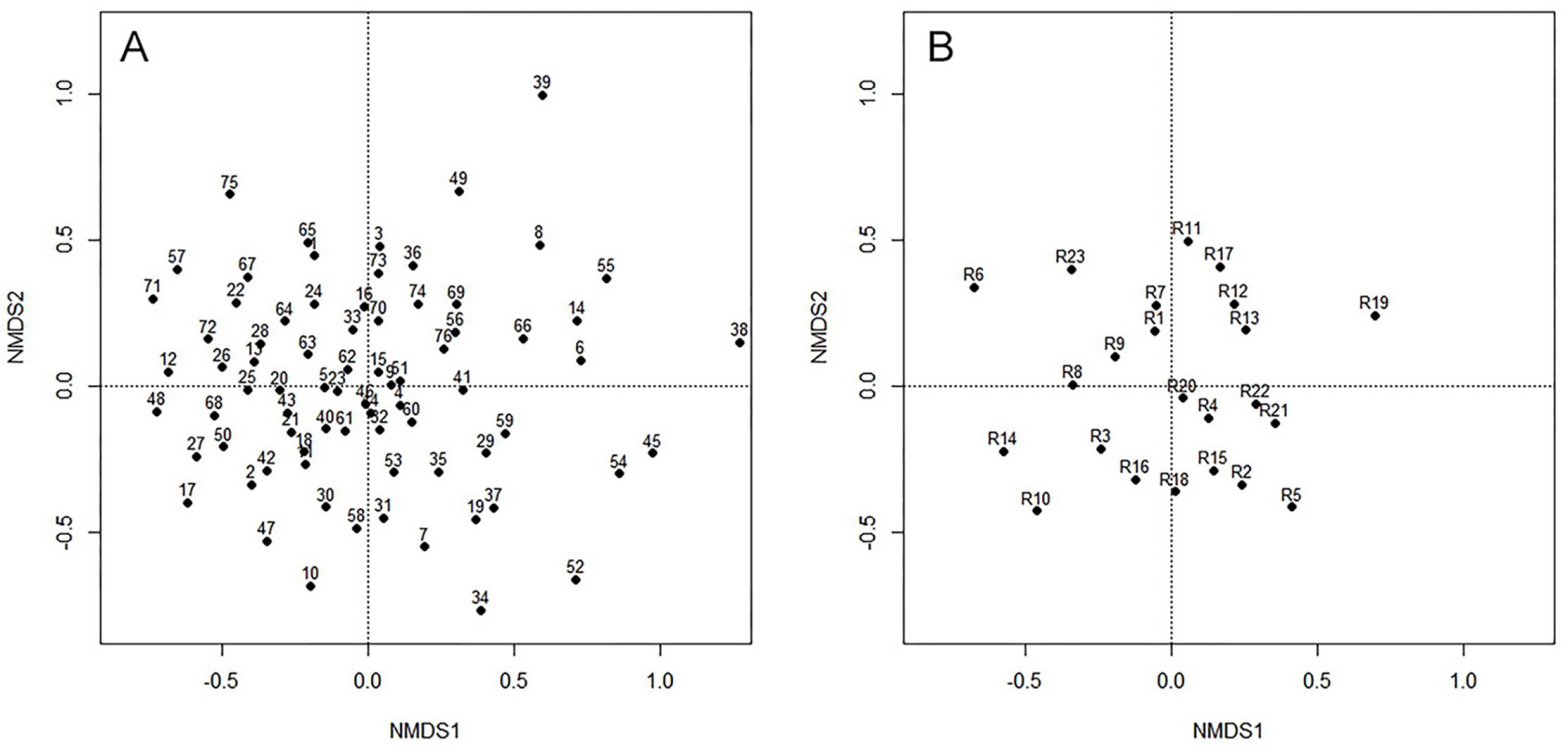
Diagrams of the Non-metric Multidimensional Analysis (NMDS) showing the absence of urban cell (plot A) and rural cell (plot B) sorting, according to similarities of species of birds in them. Codes beside black dots indicate location of urban and rural cells (see Fig 1).

Variation in the measured urban variables was successfully summarized in three principal components (Table 1), mainly correlated with impervious and guadual surface area (PC1), distance to the boundaries of the city, and the amount of open area (PC2), and forest surface area (PC3). Bird species diversity at order q = 0 decreased with noise intensity (β = -1.488; z = -3.973; p < 0.0001), increased with PC1 (β = 0.109; z = 3.3626; p < 0.001) and PC3 (β = 0.101; z = 3.169; p = 0.0015), and was unrelated to PC2 (β = -0.058; z = -1.847; p = 0.064) (Fig 5A–5D). Bird species diversity at order q = 1 also decreased with noise intensity (β = -1.64; z = -3.481; p <0.001), and increased with PC1 (β = 0.158; z = 4.049; p< 0.0001) and PC3 (β = 0.096; z = 2.432; p = 0.015) but was unrelated to PC2 (β = -0.029; z = -0.76; p = 0.4471) (Fig 5E–5H). Similarly, bird species diversity at order q = 2 decreased with noise intensity (β = -1.535; z = -2.852; p = 0.004), and increased with PC1 (β = 0.161; z = 3.572; p < 0.001) and PC3 (β = 0.094; z = 2.069; p = 0.038), but was unrelated to PC2 (β = -0.017; z = -0.394; p = 0.693) (Fig 5K–5L). When we explored the data using an NMDS analysis, we did not find an evident grouping of particular urban cells according to composition of bird species; that is, a given set of species could be present in any place of the city (Fig 6A).

**Table 1:** Principal component analysis summarizing variation in urban and rural (values in parenthesis) habitat characteristics. The highest loadings for each principal component was 0.7.

Urban and rural habitat variables	Principal Component		
	PC1	PC2	PC3
Guadual surface area (%)	0.90 (0.39)	0.12 (-0.24)	-0.23 (0.73)
Impervious surface area (%)	-0.83 (0.16)	0.40 (0.74)	-0.39 (-0.08)
Distance to the city boundary (m)	0.08 (-0.39)	0.88 (0.20)	0.12 (0.74)
Open area (%)	0.54 (0.89)	-0.70 (-0.02)	0.11 (-0-09)
Forest surface area (%)	-0.03 (-0.05)	0.07 (0.90)	0.98 (0.05)
Plantation surface area (%)*	(-0.05)	(0.90)	(0.05)
Eigenvalue	2.21 (2.11)	1.16 (1.43)	1.04 (1.11)
% of variance explained	44.14 (29.96)	23.14 (28.92)	20.87 (18.57)

* Plantation surface area was absent in urban cells.

**Fig 7:**
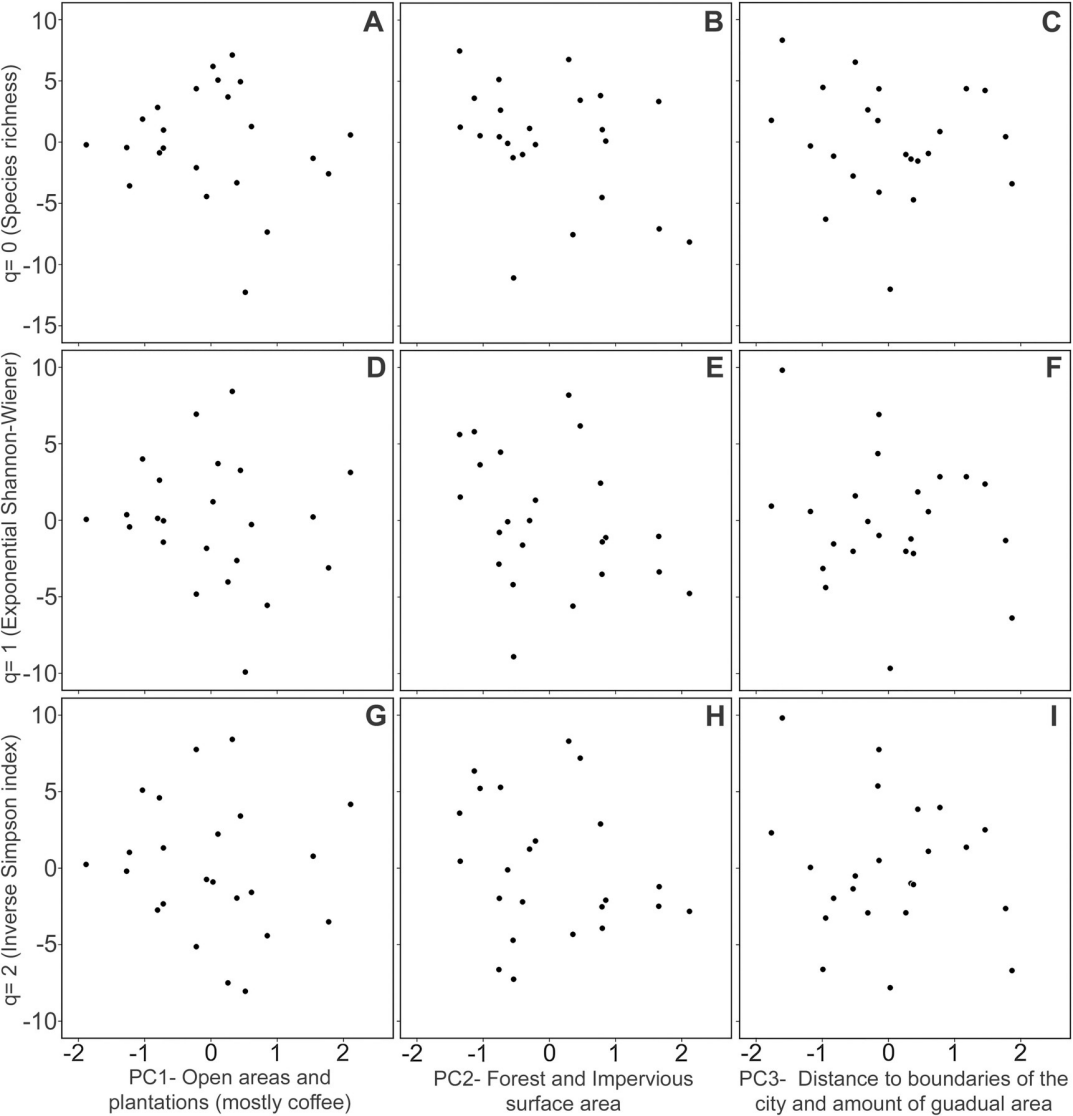
Partial plots of a multiple regression analysis showing the absence of a significant relationship between the bird species diversity at different q orders (i.e. 0,1,2) with the percentage of open and plantation surface area (A,D,G), the percentage of forested and impervious surface (B,E,H), distance to the edge of the city and guadual surface area (C,F,I), in 23 rural cells in the study area. Similar to the Fig 5, this figure has illustrative aim due that results and discussion are based on results obtained in a Generalized lineal model(GLM), however, results obtained in the GLM and the multiple regression analyses were similar. Values on *y*-axis (left) and *x*-axis (bottom) are residuals of a multiple regression analysis which means that each plot shows the net relationship between both variables after statistically controlling by the effect of the others.

In the rural habitat, variation in the measured habitat variables was summarized in three principal components (Table 1), mainly correlated with the amount of open and plantation surface area (PC1), forest and impervious surface area (PC2), and distance to the boundaries of the city and guadual surface area (PC3). Bird species diversity was not related with any of the measured habitat characteristics (Fig 7; see S1 Table for statistical results of multiple regressions). Similar to the urban habitat, we did not find a grouping pattern of particular rural cells according to the composition of bird species in them (Fig 6B).

## Discussion

According to our first objective and prediction, there was higher bird species diversity in the rural habitat than in the urban habitat; species composition differed between those habitats, yet species turnover (beta-diversity) between them was moderate. Regarding to our second objective and prediction, we found spatial variation in bird species diversity inside the urban habitat, but not in the rural habitat. Inside the city of Armenia, bird diversity increase towards places with lower levels of urbanization (i.e. those with lower noise intensity, lower impervious surface area, and higher guadual and forest surface area). Finally, we found an opposite result to that expected for the third objective; that is, both in the urban and rural habitats, there was a similar species composition among sites with similar or dissimilar habitat characteristics.

Higher diversity in rural habitats compared to urban habitats is a widespread pattern found across taxa in both tropical and temperate regions [27, 82–86]. This pattern has been attributed to a reduction in diversity and quality of resources in urban areas [32]. For instance, it is common for native plant species to be replaced by introduced species used for urbanistic ornamentation [4, 26]. These species do not attract the same quantity of insects neither produce fruits that conform the historical diet of native birds, hence leading to species diversity loss [33, 87]. In Armenia, examples of replacements of such introduced plant species occur when *Agapanthus orientalis*, *Rhododendron indicum*, *Pachystachys lutea*, *Hibiscus rosa-sinensi*, *Brunfelsia pauciflora* are used as road separators, in gardens, and in parks; however, we do not know about studies testing whether these species offer similar resources to birds than native plants in our study area. At this point, it is important to highlight that the diversity of birds was higher in rural habitats than urban habitats in spite that we sampled more months in the latter.

In Armenia, we found that the species with the highest individual counts (see Fig 2 and table in S1 Table) were those commonly observed in urban and rural disturbed habitats of Colombia, and were particularly abundant in highly urbanized cells. However, the mean number of total individuals for all species per urban cell was not different than that of rural cells. This result is contrary to what is usually reported in highly urbanized cities, where higher abundances of individuals are found in urban habitats rather than in rural ones [26, 88]. Possibly our results did not reflect that generalized tendency because most of the sampled urban cells include green areas that may ameliorate the abundance of exploiter species. This hypothesis found support in a positive and significant correlation between percentage of impervious area in urban cells and number of individuals recorded there (unpublished data).

In spite of the significant reduction in species diversity in the city, many of the species found in the urban cells were the same as those recorded in the rural cells. In both urban and rural cells, we commonly found generalist and opportunistic species that eat insects, fruits and seeds, and which nest in disturbed sites and secondary forests (e.g. *Theristicus caudatus*, *Picumnus granadendis*, and *Psittacara wagleri* [62, 89]). The moderate-to-low species turnover we found between the urban and the rural habitat may be explained by three non-mutually exclusive aspects: Armenia (1) is dominated by open perturbed areas with small, isolated patches of forest remnants in rural areas, (2) has numerous forest remnants and Neotropical giant bamboo (*Guadua angustifolia*) patches, and (3) has relatively short distances between rural areas, urban forests, and urbanized places. It is unsurprising that species turnover rates between urban and rural habitats are inversely related to the intensity of urbanization and the distance to source habitats [11,27]. Therefore, our results suggest that green areas embedded in Armenia lessen the negative effects of urbanization on bird species that tolerate some level of anthropogenic disturbance.

The moderate-to-high level of similarity in species composition between biotic assemblages in urban and rural habitats in our study area could also be influenced by processes acting at larger spatio-temporal scales that frequently are overlooked (see [6]). For instance, in temperate regions, greater temperature seasonality and the concomitant variation in the availability of favorable resources results in species with broader physiological tolerances and capacities to deal with harsher habitats than species in tropical regions [90–92]. Hence, we expect higher species turnover rates between urban and rural habitats in assemblages of tropical regions than in assemblages of temperate regions. Testing this hypothesis is beyond the scope of our study, and at present it is complicated because most analysis at broader scales are centered on species richness and relative abundances rather than species compositions [27].

Our finding of lower bird diversity in urban cells with higher noise intensity than in those with lower noise intensity is parallel to patterns found in other studies [93–95]. Noise can restrict the establishment of many species of birds in urbanized habitats through several non-exclusive factors that could reduce the fitness of individuals and populations. For instance, birds use songs for attracting mates and breeding, but anthropogenic noise can prevent intraspecific recognition via inadequate detection of acoustic signals [96–98]. Noise can also distract individuals and make them less efficient at foraging, detecting potential predators, or even, restrict their communication between parents and offspring [99, 100]. In addition, noise can increase individual stress levels, which may affect aspects such as immune responses [101]. These ecological effects of noise are not exclusive to birds [102–104].

Higher species richness in urban cells where there is higher percent coverage of green areas (forest, guadual), but lower impervious area, is an expected pattern according to the ecological requirements of birds. In green areas, many species may find resources that are less commonly found in impervious areas (e.g. nesting places, dietary items, and shelter microhabitats to biotic and abiotic environment features [33, 105, 106]). This may be the case for species such as *Momotus aequatorialis*, *Aulacorhynchus haematopygus*, and *Aramides cajanea* [107–110]. On the contrary, some species may be overly abundant in strictly impervious areas. These species tend to be very efficient at exploiting specific resources associated with human habits and anthropogenic infrastructure (i.e. *Troglodytes aedon*, *Pyrocephalus rubinus*, *Pygochelidon cyanoleuca*, *Pitangus sulphuratus*, *Myiozetetes cayanensis*, *Tyrannus melancholicus*; [63,111], (andpersonal observations).

We found that trends of higher bird diversity in forested areas was significant at order q = 0, 1 and 2 (Fig 5H and 5L). A forest remnant in an urban cell may allow the presence of additional species of birds dependent on forested habitats and hence increase diversity. Given that forest remnants are embedded in the urban matrix (with the concomitant anthropogenic perturbation), those species could be represented by very few individuals or could have been overlooked. Examples of these species include *Accipiter striatus*, *Eupsittula pertinax* and *Myioborus miniatus* [62, 112–114]. The absence of a relationship between the amount of open areas and the distance to city boundaries is attributed to the following non- exclusive reasons: (1) birds may avoid open areas because of higher predation risks; (2) open areas are structurally homogeneous and do not offer abundant resources [4]; (3) Armenia’s landscape configuration includes many areas of forest remnants, and they may influence the spatial pattern of bird species diversity more than the green areas beyond the city boundaries [15, 115].

The landscape around the city of Armenia is highly disturbed and large forested areas have been destroyed for the establishment of monoculture crops and grasslands; hence, like in urban cells, most of the bird diversity in the rural cells is composed by generalist species that are adapted to live and reproduce in open and disturbed areas [116, 117]. It is possible that the absence of any relationship between habitat characteristics and bird diversity in rural habitat could be because those species can move easily, and exploit resources associated, among grasslands, coffee crops, guadual and small forest patches that still persist there [118]. In urban cells, species could be more restrained to do that because diverse aspects associated to urbanization, for example, the presence of novel interespecific interactions (e.g. predation, parasitism) or because they tend to avoid places with high and constant abiotic noise [93, 95, 119]. In fact, the predominance of generalist species in our study also would explain the low species turnover (beta diversity) in bird diversity between urban and rural habitats. Altogether, results in this study are in accord with the negative effect of deforestation in species diversity that has been reported in the Andes of Colombia [120, 121], but also support the perception that urbanization implies stronger effects on biodiversity than other human activities (e.g. farming) because it creates novel habitats with more extreme anthropogenic conditions than animals would have experienced in the past [119, 122].

The discriminant analysis did not show a clear segregation of particular urban cells (and rural cells) according to bird species composition (i.e. urban cells tend to share many species). This result was surprising because based on the literature [11, 23] we expected to find significant differences between urban cells with green areas and those in the core of Armenia, characterized by higher percentages of impervious areas (e.g. cells 45, 56, 57 in Fig 1). Nevertheless, it is important to note that our bird surveys were made in non-forested areas, which reduced the probability of recording species in the forest interiors. For instance, in a forest remnant located at the north part of Armenia, a previous study [107] has recorded more than 20 bird species of forest interior that were undetected in our study. We were aware of this systematic bias, but for this study we were not able to perform surveys inside forest remnants within Armenia. When those samplings inside urban forests are feasible, we would expect to find significant differences in species composition between places (i.e. urban cells) with high and low levels of urbanization. Something similar could be expected for the rural habitat.

In summary, our results highlight the importance of the presence of green areas (e.g. forest and guadual remnants) in cities and towns to increase local bird diversity in assemblages. A similar conclusion was reached by [123] for amphibians and reptiles in our study city, and by other authors in studies performed with diverse taxa in both, temperate and tropical regions (see reviews by [27, 124]). This observation is especially relevant for Colombia and the Northern Andes in general, a hotspot of biodiversity, since many species exhibit narrow geographical ranges of distribution [66, 67, 125, 126]. However, how much urban green areas can ameliorate the ecological impacts of urbanization on biodiversity (and the associated ecosystem services) depends on public policies for urban development [5, 127]. If spatial variation effects on diversity patterns of birds, which are vertebrates with high dispersal capacity, were detectable at the scale of our study, there are likely similar or even stronger effects of urbanization on other animals with lower vagility. We suggest that more studies in the Northern Andes of South America and the Neotropics are necessary to understand patterns and drivers of biodiversity in relation to urbanization.

## Supporting information

S1 Table Number of individuals per species recorded in 76 urban and 23 rural cells in the city of Armenia, Central Andes of Colombia, South America (see Fig 1 and text for details). (DOCX)
