# Supplementary material for: Birds vs bricks: Patterns of species diversity in response to urbanization in a Neotropical Andean city
Source: PLoS One. 2019 Jun 20;14(6):e0218775. doi: 10.1371/journal.pone.0218775 (PMC6587937; doi:10.1371/journal.pone.0218775)
Supplement: S1 Table — (DOCX) [file pone.0218775.s001.docx]

Table S1. Number of individuals per species recorded in 76 urban and 23 rural cells in the city of Armenia, Central Andes of Colombia, South America (see Fig. 1 and text for details).

| **Taxa** | **Habitat** | |
| --- | --- | --- |
|  | **Urban** | **Rural** |
| *Accipiter striatus* | 1 | 0 |
| *Amazilia saucerrottei* | 14 | 6 |
| *Amazilia tzacatl* | 39 | 21 |
| *Anthracothorax nigricollis* | 16 | 2 |
| *Aramides cajanea* | 1 | 0 |
| *Aulacorhynchus haematopygus* | 0 | 4 |
| *Brotogeris jugularis* | 6 | 2 |
| *Bubulcus ibis* | 3 | 8 |
| *Cacicus cela* | 0 | 3 |
| *Camptostoma obsoletum* | 0 | 1 |
| *Caracara cheriway* | 1 | 1 |
| *Carduelis psaltria* | 14 | 0 |
| *Catharus aurantiirostris* | 2 | 0 |
| *Cercomacra nigricans* | 0 | 1 |
| *Chaetocercus mulsant* | 0 | 3 |
| *Chlorophanes spiza* | 1 | 2 |
| *Chlorostilbon mellisugus* | 0 | 1 |
| *Coereba flaveola* | 66 | 18 |
| *Colaptes punctigula* | 0 | 2 |
| *Colaptes rubiginosus* | 3 | 0 |
| *Columba livia* | 133 | 0 |
| *Columbina talpacoti* | 370 | 31 |
| *Contopus cinereus* | 1 | 0 |
| *Coragyps atratus* | 246 | 54 |
| *Cranioleuca erythrops* | 2 | 0 |
| *Crotophaga ani* | 12 | 19 |
| *Cyanocorax yncas* | 0 | 3 |
| *Dacnis hartlaubi* | 0 | 1 |
| *Dryocopus lineatus* | 2 | 0 |
| *Elaenia flavogaster* | 4 | 3 |
| *Elaenia frantzii* | 0 | 1 |
| *Euphonia laniirostris* | 28 | 13 |
| *Eupsittula pertinax* | 2 | 0 |
| *Florisuga mellivora* | 1 | 0 |
| *Forpus conspicillatus* | 74 | 12 |
| *Grallaria guatimalensis* | 0 | 1 |
| *Henicorhina leucosticta* | 3 | 1 |
| *Icterus chrysater* | 0 | 2 |
| *Lepidocolaptes lacrymiger* | 0 | 2 |
| *Lepidocolaptes souleyetii* | 3 | 1 |
| *Lochmias nematura* | 2 | 0 |
| *Lonchura malacca* | 0 | 2 |
| *Machetornis rixosa* | 1 | 2 |
| *Melanerpes formicivorus* | 8 | 14 |
| *Melanerpes rubricapillus* | 9 | 4 |
| *Milvago chimachima* | 10 | 4 |
| *Mionectes oleagineus* | 2 | 1 |
| *Molothrus bonariensis* | 43 | 15 |
| *Momotus aequatorialis* | 1 | 5 |
| *Myioborus miniatus* | 0 | 1 |
| *Myiozetetes cayanensis* | 50 | 20 |
| *Patagioenas cayennensis* | 9 | 4 |
| *Phaeothlypis fulvicauda* | 16 | 0 |
| *Phaethornis guy* | 0 | 1 |
| *Phimosus infuscatus* | 10 | 11 |
| *Picumnus granadensis* | 2 | 2 |
| *Pionus chalcopterus* | 10 | 6 |
| *Pionus menstruus* | 103 | 98 |
| *Piranga rubra* | 2 | 4 |
| *Pitangus sulphuratus* | 88 | 32 |
| *Polioptila plumbea* | 0 | 1 |
| *Psittacara wagleri* | 3 | 12 |
| *Pygochelidon cyanoleuca* | 790 | 45 |
| *Pyrocephalus rubinus* | 72 | 22 |
| *Ramphocelus dimidiatus* | 0 | 1 |
| *Rupornis magnirostris* | 11 | 6 |
| *Saltator atripennis* | 0 | 4 |
| *Saltator striatipectus* | 13 | 12 |
| *Sayornis nigricans* | 1 | 1 |
| *Setophaga pitiayumi* | 0 | 1 |
| *Sicalis flaveola* | 335 | 40 |
| *Spinus xanthogastrus* | 22 | 11 |
| *Sporophila intermedia* | 0 | 1 |
| *Sporophila minuta* | 2 | 1 |
| *Sporophila nigricollis* | 27 | 12 |
| *Sporophila schistacea* | 2 | 11 |
| *Stelgidopteryx ruficollis* | 3 | 5 |
| *Synallaxis albescens* | 5 | 8 |
| *Synallaxis azarea* | 0 | 1 |
| *Synallaxis brachyura* | 4 | 3 |
| *Tangara arthus* | 0 | 1 |
| *Tangara cyanicollis* | 6 | 11 |
| *Tangara gyrola* | 10 | 9 |
| *Tangara vitriolina* | 11 | 14 |
| *Tapera naevia* | 4 | 12 |
| *Taraba major* | 1 | 0 |
| *Thamnophilus multistriatus* | 10 | 9 |
| *Theristicus caudatus* | 13 | 11 |
| *Thraupis episcopus* | 224 | 112 |
| *Thraupis palmarum* | 36 | 15 |
| *Tiaris olivaceus* | 7 | 16 |
| *Todirostrum cinereum* | 15 | 11 |
| *Troglodytes aedon* | 85 | 32 |
| *Turdus ignobilis* | 63 | 37 |
| *Tyrannus melancholicus* | 63 | 20 |
| *Vanellus chilensis* | 3 | 15 |
| *Volatinia jacarina* | 4 | 14 |
| *Zenaida auriculata* | 345 | 53 |
| *Zimmerius chrysops* | 23 | 31 |
| *Zonotrichia capensis* | 0 | 7 |
